# PIK3CA-mediated PI3-kinase signalling is essential for HPV-induced transformation *in vitro*

**DOI:** 10.1186/1476-4598-10-71

**Published:** 2011-06-10

**Authors:** Florianne E Henken, N Sanjib Banerjee, Peter JF Snijders, Chris JLM Meijer, Johanna De-Castro Arce, Frank Rösl, Thomas R Broker, Louise T Chow, Renske DM Steenbergen

**Affiliations:** 1 Department of Pathology, Unit of Molecular Pathology, VU University Medical Center, PO Box 7057, 1007 MB Amsterdam, The Netherlands; 2 Department of Biochemistry and Molecular Genetics, University of Alabama at Birmingham, Birmingham, Alabama 35294-0005, USA; 3 Division of Viral Transformation Mechanisms, German Cancer Center, Im Neuenheimer Feld 242,D-69120 Heidelberg Germany

## Abstract

**Background:**

High-risk human papillomavirus (hrHPV) infections are causally related to cervical cancer development. The additional (epi)genetic alterations driving malignant transformation of hrHPV-infected cells however, are not yet fully elucidated. In this study we experimentally assessed the role of the PI3-kinase pathway and its regulator PIK3CA, which is frequently altered in cervical cancer, in HPV-induced transformation.

**Methods:**

Cervical carcinomas and ectocervical controls were assessed for PIK3CA mRNA and protein expression by quantitative RT-PCR and immunohistochemical staining, respectively. A longitudinal *in vitro *model system of hrHPV-transfected keratinocytes, representing the immortal and anchorage independent phenotype, was assayed for PI3-kinase activation and function using chemical pathway inhibition i.e. LY294002 treatment, and PIK3CA RNA interference. Phenotypes examined included cellular viability, migration, anchorage independent growth and differentiation. mRNA expression of hTERT and HPV16 E6E7 were studied using quantitative RT-PCR and Northern blotting.

**Results:**

Cervical carcinomas showed significant overexpression of PIK3CA compared to controls. During HPV-induced transformation *in vitro*, expression of the catalytic subunit PIK3CA as well as activation of downstream effector PKB/AKT progressively increased in parallel. Inhibition of PI3-kinase signalling in HPV16-transfected keratinocytes by chemical interference or siRNA-mediated silencing of PIK3CA resulted in a decreased phosphorylation of PKB/AKT. Moreover, blockage of PI3-kinase resulted in reduced cellular viability, migration, and anchorage independent growth. These properties were accompanied with a downregulation of HPV16E7 and hTERT mRNA expression. In organotypic raft cultures of HPV16- and HPV18-immortalized cells, phosphorylated PKB/AKT was primarily seen in differentiated cells staining positive for cytokeratin 10 (CK10). Upon PI3-kinase signalling inhibition, there was a severe impairment in epithelial tissue development as well as a dramatic reduction in p-PKB/AKT and CK10.

**Conclusion:**

The present data indicate that activation of the PI3-kinase/PKB/AKT pathway through PIK3CA regulates various transformed phenotypes as well as growth and differentiation of HPV-immortalized cells and may therefore play a pivotal role in HPV-induced carcinogenesis.

## Background

It has been well established that high-risk human papillomavirus (hrHPV) infections are causally related to cervical cancer development [1]. While the two most potent viral oncogenes E6 and E7 are necessary for the initiation and maintenance of cellular proliferation [2,3], their expression is not sufficient for full transformation of epithelial cells. Hence, there are extensive efforts towards identifying additionally required host cell events. Chromosomal analysis has revealed that a gain of chromosome 3q is the most common event in the development of cervical squamous cell carcinoma (SCC) [4-6]. In fact, a gain of chromosome 3q was found in all SCC previously analysed by microarray CGH [6]. Candidate oncogenes on chromosome 3q include the gene encoding p110α, the active subunit phosphatidylinositol 3-kinase catalytic alpha (PIK3CA) of class I PI3-kinase. Upon activation, PI3-kinase initiates events leading to phosphorylation of PKB/AKT, which affects additional downstream signalling proteins involved in survival and cell growth. Indeed, deregulation of the PI3-kinase pathway is common in many human malignancies [7]. In cervical carcinomas, an increased copy number of PIK3CA was positively correlated with an increase in phosphorylated PKB/AKT, one of the downstream effectors [8]. Additionally, the level of p-PKB/AKT expression increased proportional to the histopathological grade of (pre)malignant cervical diseases [9,10]. Although it has been found that HPV16E7 can activate PKB/AKT in differentiating cells [10], the relevance of PI3-kinase signalling in the process of cervical cancer development following a transforming hrHPV infection remains to be experimentally explored. Moreover, no functional studies on the specific role of PIK3CA in cervical carcinogenesis have yet been performed.

Previously we have shown that *in vitro *transformation of primary keratinocytes mediated by full-length hrHPV was accompanied with a gain of chromosome 3q in immortalized descendants [6]. This model system of HPV-transformed keratinocytes therefore provides interesting and useful source material to study the potential functional role of PI3-kinase for the various transformed phenotypes. In the present study we analysed PIK3CA expression in cervical squamous cell carcinomas. We also performed functional analyses of the contribution of PI3-kinase signalling, and specifically PIK3CA, to hrHPV-mediated transformation *in vitro*.

## Methods

### Cell culture, LY294002 treatment and transfection

Primary human foreskin keratinocytes, HPV16 and HPV18-immortalized keratinocyte cell lines (FK16A and FK18A) as well as HPV16E6E7 containing keratinocytes were cultured as described previously [11]. The latter cells were generated by transduction of primary human foreskin keratinocytes with the retroviral vector pLZRSneo containing HPV16E6E7, as described previously [12].

FK16A cells between passages 45 and 62 represented immortal and anchorage dependent cells and FK16A cells between passages 99 and 189 represented anchorage independent cells [11]. Prior to LY294002 (10 μM and 20 μM) (Cell Signaling Technology, Beverly, USA) or DMSO treatment, cells were starved overnight to ensure similar phosphorylation status. A pool of 4 siRNA sequences targeting PIK3CA (cat#L-003018-00-0005, Dharmacon, Lafayette, USA) was transfected using Dharmafect reagent 2 (Dharmacon) according to the manufacturers protocol. Pools of 4 non-targeting siRNAs (cat#D-001810-10-05) and PLK1 specific siRNAs (cat#L-003290-00-0005) were used as negative and positive controls, respectively. The use of a non-targeting siRNA pool ensures control for off-target effects. Transfection of cDNA encoding for myristoylated PIK3CA [13] Addgene, Cambridge, USA) and cotransfections with HPV16-URR luciferase constructs into FK16A cells were performed using Effectene (Qiagen, Hilden, Germany) according to instructions. Firefly luciferase and Renilla luciferase were measured using Dual Luciferase assay (Promega, Wisconsin, USA).

### Clinical material

All tissue specimens were collected during the course of routine clinical practice at the Department of Obstetrics and Gynecology at the VU University medical center. Normal epithelial control samples were obtained from histologically normal frozen biopsies of non-cancer patients undergoing hysterectomy. This study followed the ethical guidelines of the Institutional Review Board of the VU University medical center.

### RNA isolation, RT-PCR and Northern Blotting

Isolation of mRNA from cell lines was performed using RNA-B reagent (Tel-Test, Friendswood, USA) and DNase treated (Promega) prior to cDNA synthesis using specific reverse primers (see below). Total RNA from micro-dissected frozen biopsies of cervical SCCs and normal ectocervical controls was isolated using Trizol reagent (Invitrogen Life Technologies, Breda, The Netherlands) as described before [14]. Quantitative RT-PCR was performed as described previously [15] using the following primers for PIK3CA forward 5'-CCTGATCTTCCTCGTGCTGCTC-3' and reverse 5'- ATGCCAATGGACAGTGTTCCTCTT -3' using SYBR Green PCR Master Mix (Applied Biosystems, Carlsbad, CA, USA). And for hTERT forward 5'-CACGCGAAAACCTTCCTCA -3', reverse 5'-CAAGTTCACCACGCAGCC-3' and the probe FAM-5'-CTCAGGGACACCTCGGACCAGGGT -3'-TAMRA using Universal PCR Master Mix (Applied Biosystems). To correct for RNA quality and input, we performed RT-PCR for the housekeeping gene snRNP as described before in cell line experiments [16]. For quantification, a standard curve was established using serial dilutions of cervical cancer cell line cDNA. To determine HPV16E7 mRNA expression LightCycler real-time PCR assays were applied as described before [17,18] as well as Northern Blotting for HPV16. Total RNA was separated on a 1% agarose gel, blotted on nylon membranes (GeneScreen, PerkinElmer Life Sciences, Waltham, USA) and hybridized with a radioactive labelled full length HPV16 probe.

### Immunoblotting

Antibodies against total (cat#9272) or phosphorylated forms of PKB/AKT (cat#4058), PIK3CA (cat#4255) and loading control beta-actin (cat#4967) (all 1:1000 from Cell Signaling Technology) were used according to the manufacturers instructions. Membranes were incubated with the appropriate horseradish peroxidase-conjugated secondary antibodies and the levels of corresponding proteins were visualized using SuperSignal West Dura Extended Duration Substrate (Pierce).

### Immunohistochemical staining and immunofluorescence assays

Immunohistochemical staining was performed using 4 μm sections which were deparaffinised, rehydrated and microwave-treated (800W) for 10 min in Tris buffer (pH9), followed by incubation for 30 min in 3% H_2_O_2 _in methanol. Antibody incubation with PIK3CA (cat#4249 Cell Signaling 1:200) was performed overnight at 4°C and for detection the EnVision horseradish peroxidase system (Dako, Heverlee, Belgium) was used.

For immunofluorescence, 4 μm sections were rehydrated, treated with 10 mM citrate, pH 6.0 at 95°C for 10 min and allowed to cool to room temperature over 20 min. Slides were then treated with 3% H_2_O_2 _in water and blocked in 1XPBS containing 10% goat serum. P-PKB/AKT and Ki-67 were detected by sequential probing with respective antibodies because both were raised in rabbit. P-PKB/AKT was probed with rabbit monoclonal antibody (cat#2118-1, Epitomics Inc, 1:100 dilution) and detected by fluorescine-conjugated tyramide (NEL701001, PerkinElmer Life Science, USA) as per the manufacturers direction. Subsequently, Ki-67 was probed with rabbit monoclonal antibody (ab16667, Abcam, 1:100 dilution) and detected by Alexa Fluor 555 conjugated anti-rabbit IgG (cat#A21429, Invitrogen-Molecular Probes, USA.) as per the manufacturers protocol. P-PKB/AKT and CK10 localization was detected by concurrent probing with respective primary antibodies as they were raised in different species. Raft sections were treated with rabbit anti p-PKB/AKT (described above) and mouse monoclonal anti-CK10 (cat#ab1421, 1:150 dilution, Abcam, Cambridge, USA). Anti-CK10 was detected with Alexa Fluor 555 conjugated goat anti-mouse IgG (cat#A21424, Invitrogen-Molecular Probes, USA). Finally slides were mounted with DAPI containing media (VECTASHIELD, H1200, Vector Laboratories, USA), viewed under Olympus AX70 microscope fitted with Chroma filters. Photomicrographs were captured by Axiovision camera at 20× magnifications of objective and finally processed with Photoshop CS2 (Adobe) for documentation.

### Cell Viability, Migration, Anchorage Independent Growth

Cell viability was assessed by MTT (3-(4,5-dimethylthiazol-2-yl)-2,5-diphenyltetrazolium bromide) dye reduction (ICN Biomedicals Inc, USA). Cells were seeded in triplicate wells in 96-wells plates, transfected or starved overnight followed by LY294002 treatment and grown for 5 days. Control conditions were set to 100%.

For cellular migration assays cells were plated at high confluence and uniformly scratched to create a cell-free gap. After 24-48 hours in serum-free keratinocyte growth medium, plates were examined and photographed to asses the migration of neighbouring cells into the gap.

To examine anchorage independent growth under the different conditions 5000 cells of each condition were plated in semi-solid agarose (as described previously [19]). After 3 weeks colonies larger than ~50 cells were counted and pictures taken.

### Organotypic raft cultures

The HPV containing keratinocytes were grown as epithelial raft tissues as described previously [20]. For all conditions duplicate rafts were developed. Transfections with siRNAs were carried out the day before seeding on the collagen beds. Inhibitor treatment was started after seeding and continued throughout the 9 days of culturing at the liquid-air interface. After harvesting, the raft tissues were fixed in formalin and embedded in paraffin. For histological examination, 4 μm sections were stained with hematoxylin and eosin.

### Statistical analysis

All statistical analyses were carried out using the T-test in the SPSS software package (SPSS 15.0, Chicago, USA).

## Results

### Increased PIK3CA expression in cervical carcinomas

**Figure 1:**
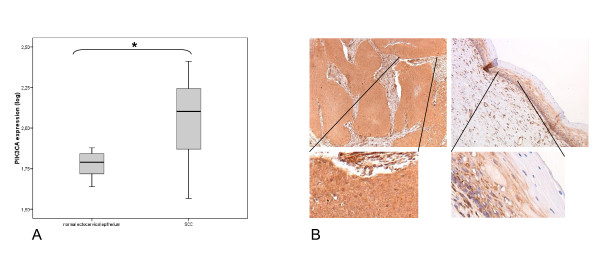
**PIK3CA expression**. (**A**) mRNA expression levels of PIK3CA in SCCs compared to normal ectocervical epithelium determined by quantitative RT-PCR. The upper and lower boundaries of the boxes represent the 75th to 25th percentile, bars within the boxes represent the median value. Whiskers represent the minimum and maximum values. (**B**) Representative pictures of immunostainings for PIK3CA on a cervical carcinoma specimen (left) and normal cervical epithelium (right) (20× magnification).

To investigate the relevance of PI3-kinase pathway activation in cervical carcinogenesis, mRNA and protein expression levels of the catalytic subunit PIK3CA were examined. As can be seen in Figure 1A messenger RNA levels for PIK3CA were significantly higher in SCC (n = 12) as compared to normal ectocervical controls (n = 10) (p = 0.005). All cervical carcinomas were previously analysed with arrayCGH and contained a gain of chromosome 3q [6], suggesting that elevated mRNA expression may be related to increased genomic content. A subset of cervical carcinomas was also analysed for PIK3CA protein expression using immunohistochemistry. Diffuse PIK3CA protein expression was observed in the tumor fields, whereas the upper strata were negative in adjacent normal cervical epithelium (Figure 1B).

### The PI3-kinase pathway becomes increasingly activated during hrHPV-induced transformation *in vitro*

To examine whether the PI3-kinase pathway is relevant for HPV-mediated transformation *in vitro*, we made use of our model system consisting of primary keratinocytes transfected with full-length HPV16. This longitudinal model consists of successive stages of transformation, including immortalization and anchorage-independent growth. The immortal cells have bypassed the senescence and crisis barriers that mark the end of the replicative and extended lifespan, respectively, and display increased telomerase activity [11]. At this stage, the cells also possess a gain of chromosome 3q [6]. Upon further passaging the immortal cells acquired an anchorage-independent phenotype [19].

**Figure 2:**
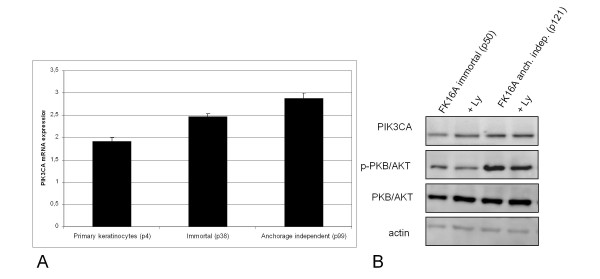
**Expression of PIK3CA, p-PKB/AKT and total PKB/AKT expression in FK16A cells**. **(A) **mRNA expression for PIK3CA in primary keratinocytes (p4), immortal FK16A cells (p38) and anchorage independent FK16A cells (p99). Shown are PIK3CA levels relative to snRNP. **(B) **Protein expression of PIK3CA, p-PKB/AKT, total PKB/AKT and actin in FK16A cells at immortal (p50) and anchorage independent (p121) stage. Second lanes for each cell line represent samples treated with 20 μM LY294002 (Ly) to inhibit PI3-kinase signalling. P: passage.

HPV16-transfected keratinocytes (FK16A) representing these two different stages of transformation were analysed for PIK3CA mRNA and protein expression. One of the downstream targets of PIK3CA activation, the phosphorylated PKB/AKT, along with total PKB/AKT were also analysed. FK16A cells showed an increase in PIK3CA mRNA expression with progression from immortal to an anchorage-independent phenotype (Figure 2A), which was associated with a slight elevation in PIK3CA protein expression (Figure 2B). During passaging a stronger increase in phosphorylation of PKB/AKT was seen (Figure 2B), indicative of a progressive activation PI3-kinase signalling. Analysis of HPV18 transfected keratinocytes derived from the same donor also revealed an increase in phosphorylated PKB/AKT with passaging (Additional File 1, Figure S1). Similarly, HPV16E6E7 containing keratinocytes obtained from a second donor displayed increasing p-PKB/AKT levels with passaging (Additional File 2, Figure S2). The latter cells also showed a slight increase in PIK3CA protein expression at later passage. These data indicate that PI3-kinase signalling becomes progressively activated during HPV-induced transformation and is donor and hrHPV type independent.

Phosphorylation and activation of PKB/AKT is a result of upstream signalling events. Hence, we proceeded to determine whether we could influence PKB/AKT phosphorylation by modulating PI3-kinase activity in our model system. Different passages of FK16A cells were treated with the chemical PI3-kinase inhibitor LY294002, which fits in the ATP binding pocket of the catalytic subunit PIK3CA [21]. LY294002 treatment resulted in reduction of the levels of phosphorylated PKB/AKT at all passages, while total PKB/AKT remained the same (Figure 2B). These results indicate that phosphorylation of PKB/AKT in these cells relies at least in part on the activity of PI3-kinase signalling.

### PI3-kinase signalling is functionally involved in hrHPV-induced transformation *in vitro*

**Figure 3:**
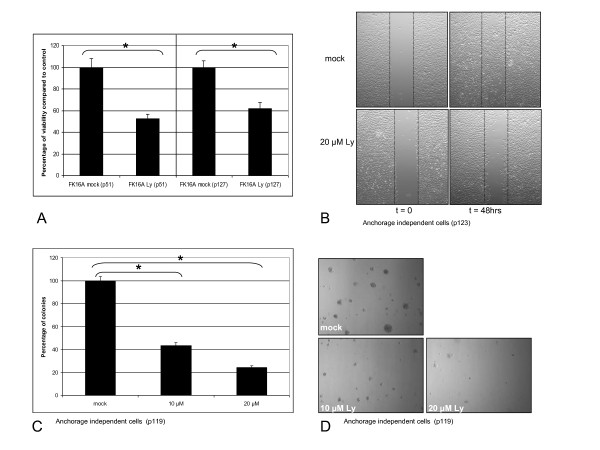
**The functional consequences of silencing PI3-kinase signalling using the chemical inhibitor LY294002 (Ly) in FK16A cells**. **(A) **Cell viability as measured 4 days post-treatment using an MTT assay. Appropriate controls were set to 100%. Immortal (p51) and anchorage independent (p127) FK16A cells were treated with the PI3-kinase inhibitor LY294002. **(B) **Representative pictures of migration assay performed with anchorage independent FK16A cells (p123). Upper panel: mock treated cells, lower panel: cells treated with 20 μM of the PI3-kinase inhibitor LY294002. Pictures were taken immediately after scratch induction and 48 hours post-induction. **(C) **Late passage FK16A cells (p119) were seeded in semi-solid agarose in the presence of the PI3-kinase inhibitor. After 3 weeks in culture colonies were quantified relative to the appropriate controls, set to 100%. * Indicates a statistical significant difference. **(D) **Representative pictures of anchorage independent growth results upon various LY294002 concentrations. P: passage.

To examine the biological role of the PI3-kinase pathway in our cell lines, we determined the functional consequences of LY294002 treatment. Figure 3A illustrates that for both immortal and anchorage-independent passages of FK16A incubation with LY294002 resulted in a statistically significant reduction in the number of viable cells using a viability assay (MTT) (p = 0.001 for each passage). Another important feature of transformation is the migratory capacity of cells. Figure 3B shows that, upon wound induction, HPV16-tranformed keratinocytes migrated and closed the wound after 2 days. In contrast, chemical inhibition of PI3-kinase in these cells reduced their migratory capacity, as cells were unable to close the wound. Lastly, we examined anchorage-independent growth. At a concentration of 10 μM of the inhibitor, colony formation was significantly inhibited compared to mock treated controls (p = 0.003) and decreased further at a concentration of 20 μM (p = 0.001) (Figure 3C and 3D). Taken together, the results suggest that PI3-kinase signalling is functionally relevant for the transformed phenotypes of hrHPV-immortalized cells.

### The catalytic subunit PIK3CA is essential for HPV-mediated transformation

**Figure 4:**
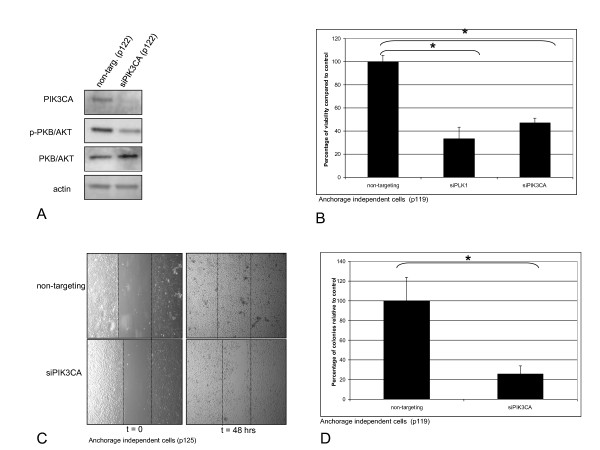
**The effect of siRNA mediated silencing of PIK3CA on protein expression, cell viability, migration and anchorage independent growth of FK16A cells**. **(A) **Protein expression of PIK3CA, p-PKB/AKT, total PKB/AKT and actin in FK16A cells (p122) transfected with non-targeting control siRNAs and siRNAs targeting PIK3CA. **(B) **Cell viability as measured 4 days post-treatment using an MTT assay. Anchorage independent (p119) FK16A cells were transfected with non-targeting control siRNAs, siRNAs targeting PIK3CA. Silencing PLK1 served as a positive control. Appropriate controls were set to 100%. * Indicates a statistical significant difference. **(C) **Representative pictures of migration assay performed with anchorage independent FK16A cells (p125). Upper panel: cells transfected with non targeting siRNA pool, lower panel: cells transfected with siRNA pool against PIK3CA. Pictures were taken immediately after scratch induction and 48 hours post-induction. **(D) **Late passage FK16A cells (p119) were seeded in semi-solid agarose following transfection with non-targeting control siRNAs or siRNAs targeting PIK3CA. After 3 weeks in culture colonies were quantified relative to the appropriate controls, set to 100%. * Indicates a statistical significant difference. P: passage.

Our findings strongly suggested a role of PIK3CA in HPV-mediated transformation. Since LY294002 is a universal PI3-kinase inhibitor affecting the different PI3-kinase classes to varied extents, we next employed RNA interference to examine PIK3CA specifically. A pool of specific siRNAs was used to silence PIK3CA expression in anchorage independent FK16A cells. Non-targeting siRNAs were used to control for off-target effects. The reduction of PIK3CA expression was confirmed by Western blotting (Figure 4A). PIK3CA silencing strongly inhibited PI3-kinase signalling as reflected by reduced phosphorylation of PKB/AKT without reduction of total PKB/AKT (Figure 4A).

Following PIK3CA specific siRNA transfection cells were seeded for the functional tests described above. Similar to LY294002 treatment, siRNA-mediated PIK3CA knockdown significantly reduced the number of viable FK16A cells (p = 0.0005) compared to transfection with a pool of non-targeting siRNAs (Figure 4B). Knock-down of PLK1, inducing cell death and used as a positive control, also resulted in a significant reduction of viable cells (p = 0.0003). FK16A cells transfected with siRNAs targeting PIK3CA also lost their migratory capacity and were unable to close the wound (Figure 4C). Primary human foreskin keratinocytes, included as controls, displayed a minor reduction in migratory capacity upon PIK3CA silencing (Additional File 3, Figure S3), in concordance with the lower level of PIK3CA mRNA in these cells (Figure 2A). Lastly, anchorage-independent growth was also affected by PIK3CA knockdown to a similar extent as after treatment with 10 μM LY294002 (p = 0.05) (Figure 4D). Taken together, these results show that PIK3CA is relevant for the oncogenic properties of HPV-mediated transformation.

**Figure 5:**
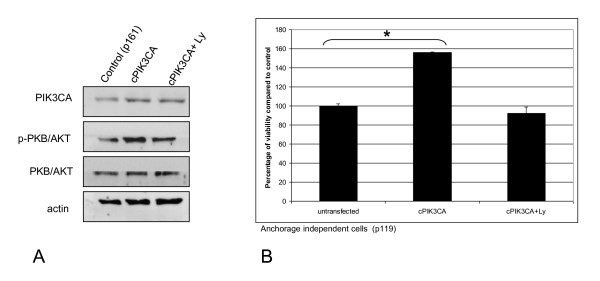
**The effect of PIK3CA overexpression in FK16A cells**. **(A) **Protein expression of PIK3CA, p-PKB/AKT, total PKB/AKT and actin in FK16A cells (p161) transfected with cDNA encoding PIK3CA as well as PIK3CA overexpressing cells treated with LY294002. **(B) **Cell viability as measured 4 days post-treatment using an MTT assay. Appropriate controls were set to 100%. * Indicates a statistical significant difference.

To substantiate our hypothesis that PIK3CA is an important mediator of PI3-kinase signalling in HPV-transformed cells, we examined the effect of PIK3CA over-activation. This was achieved by transfection of anchorage independent FK16A cells with cDNA encoding myristoylated PIK3CA in which a coding sequence for the myristoylation domain of c-Src is fused in-frame with the PIK3CA coding sequence [13]. The amino-terminal myristoylation modification ensures membrane targeting of PIK3CA, allowing PIP3 production without the otherwise required receptor activation. Figure 5A shows that transfection of PIK3CA cDNA resulted in increased levels of PIK3CA protein and also enhanced levels of p-PKB/AKT. After treatment with LY294002, levels of p-PKB/AKT reverted, whereas levels of PIK3CA remained elevated. Viable cells increased significantly in FK16A cultures with ectopically activated PIK3CA when compared with untransfected cells (p = 0.04). This effect was reversed by incubation with 20 μM LY294002 (Figure 5B). These findings further strengthen the notion of a role of PIK3CA and PI3-kinase signalling in HPV-containing cells.

### PI3-kinase signalling is involved in transcriptional regulation of HPV

**Figure 6:**
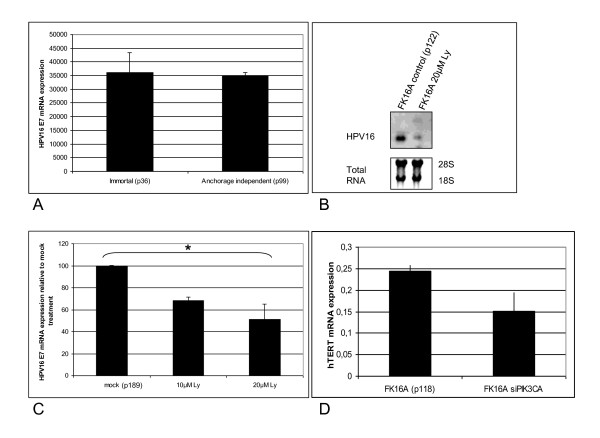
**Relation between PI3-kinase activity and HPV oncogene expression**. **(A) **HPV16E7 mRNA expression in FK16A cells at immortal (p36) and anchorage independent (p99) stage as determined by qRT-PCR. **(B) **HPV16 mRNA expression in anchorage independent FK16A cells (p122) after PI3-kinase inhibition determined by Northern blot using an HPV16 radio-labeled probe. **(C) **HPV16E7 mRNA expression determined by qRT-PCR after PI3-kinase inhibition relative to mock treatment (p189). * Indicates a statistical significant difference. **(D) **hTERT mRNA expression determined by qRT-PCR in anchorage independent FK16A cells (p118) silenced for PIK3CA compared to FK16A cells transfected with non-targeting siRNAs. P: passage.

In search of an explanation for the altered growth properties of the HPV16 containing keratinocytes upon manipulation of the PI3-kinase pathway, HPV16 expression was examined. Figure 6A shows that there was only a slight variation with increased passaging in HPV16 mRNA expression. After PI3-kinase inhibition in anchorage independent cells however, we found that HPV16 mRNA expression was strongly reduced as can be seen in the Northern Blot results in Figure 6B. Further quantification by real time RT-PCR revealed that HPV16E7 mRNA levels were significantly reduced after chemical inhibition to about half of the original expression at a concentration of 20 μM LY294002 (p = 0.04) (Figure 6C). To analyze further downstream effects of reduced HPV expression transcription of hTERT was monitored, which is known to be stimulated by E6 expression [22]. Cells in which PIK3CA was silenced and HPV mRNA expression was reduced also displayed a trend towards reduced hTERT mRNA expression (Figure 6D). Although E7 mRNA expression levels in immortal and anchorage independent cells were comparable, while PIK3CA expression and PI3-kinase activity were increased in anchorage independent cells, the results shown in Figure 6B-6D suggest a functional cross-talk between PI3-kinase and maintenance of HPV oncogene expression.

### PI3-kinase signalling is essential for growth and differentiation of HPV-immortalized cells in organotypic cultures

Culturing human keratinocytes on organotypic raft mimics epithelial stratification and differentiation into various strata, whereby PI3-kinase signalling is known to play a role [10,23-25]. We have previously shown that differentiation is disturbed in HPV16- and HPV18- immortalized keratinocytes, revealing phenotypes reminiscent of HPV-induced dysplastic lesions [20]. Thus, we next studied the roles of PI3-kinase signalling in growth and differentiation of these HPV-immortalized keratinocytes in organotypic raft cultures. FK16A cells displayed a severely dysplastic morphology on epithelial raft cultures, whereas an HPV18 immortalized cell line, FK18A, appeared less dysplastic and maintained terminal differentiation [20]. These different attributes permit a comparison of cell growth and differentiation upon modulating PI3-kinase. As described above, FK18A cells also exhibited increased levels of phosphorylated PKB/AKT with passaging (Additional File 1, Figure S1).

**Figure 7:**
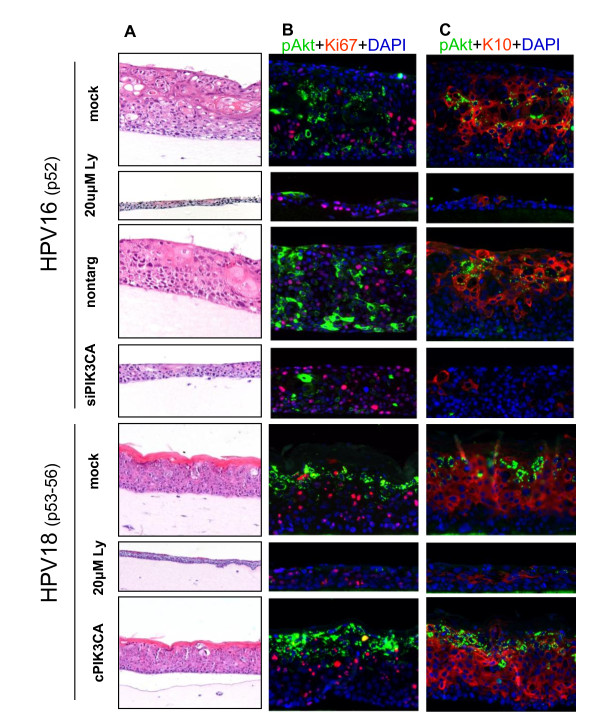
**Organotypic raft cultures of HPV16 and HPV18 immortalized keratinocytes**. After culturing in the presence or absence of PI3-kinase inhibitor LY294002, following siRNA transfection (p53) (nontarg: non-targeting siRNA; siPIK3CA: PIK3CA specific siRNAs) and upon PIK3CA overexpression (p52-56) (cPIK3CA). **(A) **H&E staining showing raft culture morphology. **(B) **Double immunofluoresence probing performed on sections of the same cultures for p-PKB/AKT (green), Ki-67 (red) and DAPI (blue) and **(C) **for p-PKB/AKT (green), CK10 (red) and DAPI (blue). P: passage.

Epithelial raft culturing of immortal FK16A cells in the presence of the PI3-kinase inhibitor or after silencing PIK3CA using siRNAs, hampered epithelial raft culture development compared to mock treated cells or cells treated with non-targeting siRNAs (Figure 7A). Particularly upon LY294002 treatment the epithelium was very thin, whereas transfection of PIK3CA-siRNAs had less effect on epithelial growth. These observations suggest that PIK3CA plays a role in the growth of these cells and that chemical inhibition of PI3-kinase most likely has additional effects besides PKB/AKT inactivation. Similarly, raft culture growth of FK18A cells was strongly reduced upon chemical inhibition of PI3-kinase. No clear histological difference was seen in FK18A cells over-expressing activated PIK3CA (Figure 7A). Analysis of primary human foreskin keratinocytes showed no effect on growth on organotypic culture upon PIK3CA silencing (see Additional File 4, Figure S4).

To further assess the relation between phosphorylated PKB/AKT, cell proliferation and squamous differentiation, we performed double indirect immunostainings for p-PKB/AKT and Ki-67, a proliferation marker, or cytokeratin 10 (CK10), a differentiation marker.

As shown in Figure 7B, in raft cultures of FK16A cells, the p-PKB/AKT signals were primarily detected in the cytoplasm, while Ki-67 was restricted to the nucleus. Both were detected stochastically throughout most of the epithelium. In the FK18A raft cultures, while Ki-67-positive cells were found in all cell strata, the p-PBK/AKT signal was primarily detected in the upper most differentiated strata. Interestingly, the two signals rarely co-localized to the same cells.

After chemical inhibition of PI3-kinase, a fraction of the remaining epithelial cells were positive for Ki-67 despite the fact that phosphorylated PKB/AKT was strongly reduced or nearly abolished. In the presence of siRNAs targeting PIK3CA, the p-PKB/AKT signals in FK16A raft cultures were also greatly reduced while Ki-67 signals were not altered. The non-targeting siRNAs had no effect on either. Upon ectopic expression of activated PIK3CA, more cells in the upper strata of the FK18A raft cultures were positive for p-PKB/AKT while the signals of Ki-67 appeared unchanged.

Figure 7C shows that, in both cell lines, differentiation marker CK10 was positive from the suprabasal layers upwards. Consistent with the high dysplastic histology (Figure 7A), CK10 expression was patchy in the FK16A raft cultures with packets of CK10-negative cells, whereas the signals were much more uniformly detected across the FK18A cultures (Figure 7C). After chemical inhibition of PI3-kinase signalling in FK16A and FK18A cultures, not only p-PBK/AKT was largely abolished, but also the fraction of CK10-positve cells and signal strengths were both greatly reduced, especially in FK16A cultures. Similarly, in the siPIK3CA-treated FK16A cultures, p-PKB/AKT was virtually abrogated while CK10 signals were greatly reduced. In contrast, the non-targeting siRNAs had no effect on either signal. These results strongly suggest that differentiation was largely abolished upon reduction of p-PKB/AKT. Despite an increased phophorylated PKB/AKT staining in FK18A cells overexpressing PIK3CA, CK10 staining patterns were similar to the non-transfected controls, because these cells were already strongly positive for CK10. These data confirm that PI3-kinase signalling is important for regulating differentiation of HPV-immortalized cells.

## Discussion

The PI3-kinase/PKB/AKT signalling pathway affects a wide variety of cellular characteristics such as proliferation, differentiation and cell survival and is often altered in many human malignancies [7]. In cervical cancer, a gain of the long arm of chromosome 3, where PIK3CA is located, is often described and suggested to be a compulsory second hit for malignant transformation following an hrHPV infection [4,6]. However, data is scarce on the biological effects of altered PIK3CA expression and PI3-kinase signalling in cervical carcinogenesis.

In the present study we showed that mRNA expression of the catalytic subunit PIK3CA was significantly upregulated in cervical SCC. In our previous studies using arrayCGH, up to 100% of cervical SCC were shown to contain additional copies of chromosome 3q [6]. Additionally, low levels PIK3CA amplifications were found in 40%-74% of cervical carcinomas [5,8,26]. Bertelsen *et al *showed that an increase in PIK3CA copy numbers (more than 3 copies) was significantly correlated to elevated p-PKB/AKT expression in cervical SCC and high-grade precursor lesions [8]. Also, other studies reported an increase in p-PKB/AKT staining with increase of histopathological grade of cervical disease [9,10].

Functionally, we showed that PIK3CA and concomitant PI3-kinase signalling is involved in the different stages of HPV-mediated transformation *in vitro*. Modulating PI3-kinase activity in HPV transfected keratinocytes affected proliferation, migration, anchorage independent growth, and epithelial growth and differentiation in organotypic cultures. These data are consistent with previous reports using different cellular systems. For instance, in ovarian cancer cell lines PI3-kinase pathway inhibition with LY294002 resulted in reduced proliferation via cell cycle arrest in G1 [27] or induction of apoptosis [28]. Proliferation measured by cell number and [^3^H]-Thymidine incorporation was also reduced in rat intestinal epithelial cells as a result of LY294002 treatment [29]. Furthermore, the involvement of PI3-kinase in colony formation has been shown in mammary epithelial cells where overexpression of PIK3CA increased colony formation while a dominant negative form of PIK3R1 (p85) lacking the PIK3CA binding domain repressed colony formation [30]. This latter study also showed that, although PKB/AKT is the main effector of PI3-kinase, PKB/AKT activation cannot substitute PI3-kinase signalling.

Functional studies in cervical cells are restricted to the common hrHPV positive cervical cancer cell lines such as SiHa, HeLa and CaSki and did not include the explicit analysis of PIK3CA as a candidate oncogene. Treatment of SiHa with the PI3-kinase inhibitor LY294002 led to reduced proliferation and increased apoptotic DNA fragments [5]. For HeLa and CaSki the reduction in proliferation upon LY294002 treatment was shown to be independent of apoptosis, though did sensitize the cells to radiation [31].

None of the previous studies on cancer cells have had the benefit of a longitudinal characterization as is afforded by our *in vitro *model system, which mimics the different stages of cervical carcinogenesis. We were able to show a progressive upregulation of PI3-kinase signalling during HPV-induced transformation by using the elevation of p-PKB/AKT as a reporter. Indeed, p-PBK/AKT was elevated in anchorage independent cells relative to HPV-immortalized cells. The expression levels of both PIK3CA and especially p-PKB/AKT in the latter cells were increased compared to primary keratinocytes (data not shown). This increased signalling functionally correlated with multiple attributes of transformed cells, such as cell growth, migration, and anchorage independent growth (Figure 3). The fact that specific silencing of PIK3CA using RNA interference affected each of the same transformed phenotypes in a negative manner, while PIK3CA overexpression increased proliferation (Figures 4 and 5) substantiates its function as an oncogene in cervical carcinogenesis, as has been suggested in previous studies [5,8,26].

Remarkably, our data also suggest a feedback effect in which PI3-kinase regulates HPV oncogene expression. HPV16 mRNA expression and hTERT mRNA were decreased after inhibition of PI3-kinase signalling (Figure 6). The downregulation of hTERT mRNA may be a direct result of reduced HPV expression, as E6 activates hTERT transcription [22]. Thus, the reciprocal regulation between PIK3CA/PI3-kinase and viral oncogene expression acts in concert in maintaining the transformed phenotypes [32-34]. However, it is presently unknown whether the phenotypical effects of PI3-kinase inhibition seen in our model system reflect a direct consequence of E6/E7 repression or vice versa.

It has been shown that the oncoprotein E7 is able to activate PKB/AKT, which appeared to be dependent on its ability to bind and inactivate Rb gene family proteins [10,35]. E7 may also maintain PKB/AKT in an active state, by binding and sequestering a known binding partner, the phosphatase PP2A, thereby inhibiting dephosphorylation of p-PKB/AKT [36]. Active PKB/AKT is also known to activate MDM2, enhancing p53 degradation and elevating CDKs leading to pRb inactivation, further enhancing the established E6/E7 effect on p53 and pRb function [37,38]. Moreover, activation of PKB/AKT by PI3-kinase can inhibit nuclear localization of p27kip1 and p21cip by phosphorylating the nuclear localization signal and hereby preventing nuclear transport and inducing proliferation, both of which are also affected by E7 overexpression [35,39]. Here we show that besides the reported effect of HPVE7 on PI3-kinase activity, there is also a feed-back effect in that PI3-kinase can regulate HPV oncogene expression. From the present data it becomes clear that both mechanisms act in concert as the increased PI3-kinase activity appears essential for maintaining HPV oncogene expression. The need for a strict regulation of HPV expression, as also implicated in our previous study [40], suggests that yet other transformation-inducing host cell alterations contribute to the regulated expression of HPVE6/E7 as well as PI3-kinase signalling activity.

Organotypic raft cultures mimic a natural environment to evaluate the role of PI3-kinase signalling in the growth and differentiation of keratinocytes. Phosphorylation of PKB/AKT appeared to be tightly linked to differentiation, which is in agreement with a previous report showing upregulation of phosphorylated PKB/AKT in organotypic raft cultures of HPV16 expressing keratinocytes [10]. Inhibiting PI3-kinase signalling by chemical inhibition and PIK3CA siRNA-mediated silencing during raft formation led to a dramatic reduction in p-PKB/AKT and severely hampered epithelial cell growth in both HPV16- and HPV18 immortalized cells (Figure 7). Proliferation marker Ki-67 remained detectable in residual epithelial cells, while differentiation marker CK10 was dramatically reduced. These results indicate that PIK3CA regulated p-PKB/AKT expression is important in squamous differentiation. This conclusion is in accordance with a previous report showing that mouse keratinocytes with active PKB/AKT have higher levels of differentiation markers Keratin 1, filaggrin and loricrin. Moreover, inhibition of PI3-kinase resulted in the specific death of differentiating keratinocytes [23]. Similarly, treatment of primary esophageal keratinocytes with LY294002 resulted in an overall decrease in the number of basal keratinocytes and thickness of the epithelium [41]. It has been suggested that PI3-kinase becomes important upon commitment of keratinocytes to differentiation, initiating the process and subsequently delivering the required survival signals [24]. Based on the histology of our raft cultures, there were no apoptotic cells that are typified by highly condense and shrunken nuclei (Figure 7A). The thinning of the epithelium appears to stem from a severely curtailed proliferation. Hence, in organotypic raft culture systems, p-PKB/AKT appears to correlate with squamous differentiation and survival but not with proliferation, as is also evident from the lack of co-localization between p-PKB/AKT and Ki-67 (Figure 7). Also in a study by Menges et al [10] p-AKT is seen in differentiated non-proliferating BrdU negative cells. These observation are also in line with previous studies showing that AKT knock-out MEFs reduce BrdU incorporation by only 44-61% [42], indicating that AKT is not indispensible for proliferation, though the lack of it would slow down cell cycle progression at G1-S. We hypothesize that the balance in the dual character of PI3-kinase/PKB/AKT signalling shifts during the process of HPV-induced transformation towards tumor characteristics, rather than differentiation.

## Conclusions

We have demonstrated that activation of the PI3-kinase/PKB/AKT pathway, in part resulting from increased PIK3CA expression, is functionally involved in HPV-mediated transformation *in vitro*. PIK3CA-mediated PI3-kinase signalling appeared essential in regulating proliferation, anchorage-independent growth, migration and importantly viral oncogene expression required to maintain the transformed phenotypes. Moreover, PIK3CA expression and PI3-kinase signalling emerged as critical regulators of epithelial growth and differentiation in organotypic raft cultures of HPV-immortalized cells. Hence, PIK3CA and/or the PI3-kinase/PKB/AKT pathway may provide suitable targets for therapeutic intervention in patients with HPV-induced carcinomas.

## Competing interests

The authors Prof dr LT Chow, Prof dr T Broker and dr NS Banerjee are funded by NIH. Other authors have no competing interests.

## Authors' contributions

FEH performed experiments and drafted the manuscript. NSB carried out the immunofluorescent stainings. RDMS, PJFS, CJLMM, LTC, TRB participated in design of the study and interpretation of data and helped to draft the manuscript with critical revisions for intellectual content. JDCA and FR participated in HPV expression analysis and critically revised the manuscript. All authors read and approved the final manuscript.

## Supplementary Material

Additional file 1 **Figure S1. Phosporylated-PKB/AKT and total PKB/AKT protein expression in HPV18-containing keratinocytes**. Protein lysates of HPV18-containing keratinocytes at passage 31 and passage 95 were analysed for p-PKB/AKT, PKB/AKT and actin protein expression by Western blot analysis. Click here for file

Additional file 2 **Figure S2. PIK3CA, phosporylated-PKB/AKT and total PKB/AKT protein expression in HPV16E6E7 containing keratinocytes from a different donor**. Lysates were analysed for protein expression at passage 13 and passage 60 for PIK3CA, p-PKB/AKT, PKB/AKT and actin. Click here for file

Additional file 3 **Figure S3. Migration assay of primary human foreskin keratinocytes without and with PIK3CA silencing**. Representative pictures of migration assay performed with primary keratinocytes (p5). Upper panel: cells transfected with non-targeting siRNA pool, lower panel: cells transfected with siRNA pool against PIK3CA. Pictures were taken immediately after scratch induction and 24 hours post-induction. Click here for file

Additional file 4 **Figure S4. Organotypic raft cultures of primary human foreskin keratinocytes without and with PIK3CA silencing**. H&E staining showing raft culture morphology following transfection of primary human foreskin keratinocytes (p3) with non-targeting siRNAs (left) and siRNAs targeting PIK3CA (right). Click here for file
