# Supplementary figures and images for: PIK3CA-mediated PI3-kinase signalling is essential for HPV-induced transformation in vitro
Source: Mol Cancer. 2011 Jun 10;10:71. doi: 10.1186/1476-4598-10-71 (PMC3130697; doi:10.1186/1476-4598-10-71)

## Slide 1
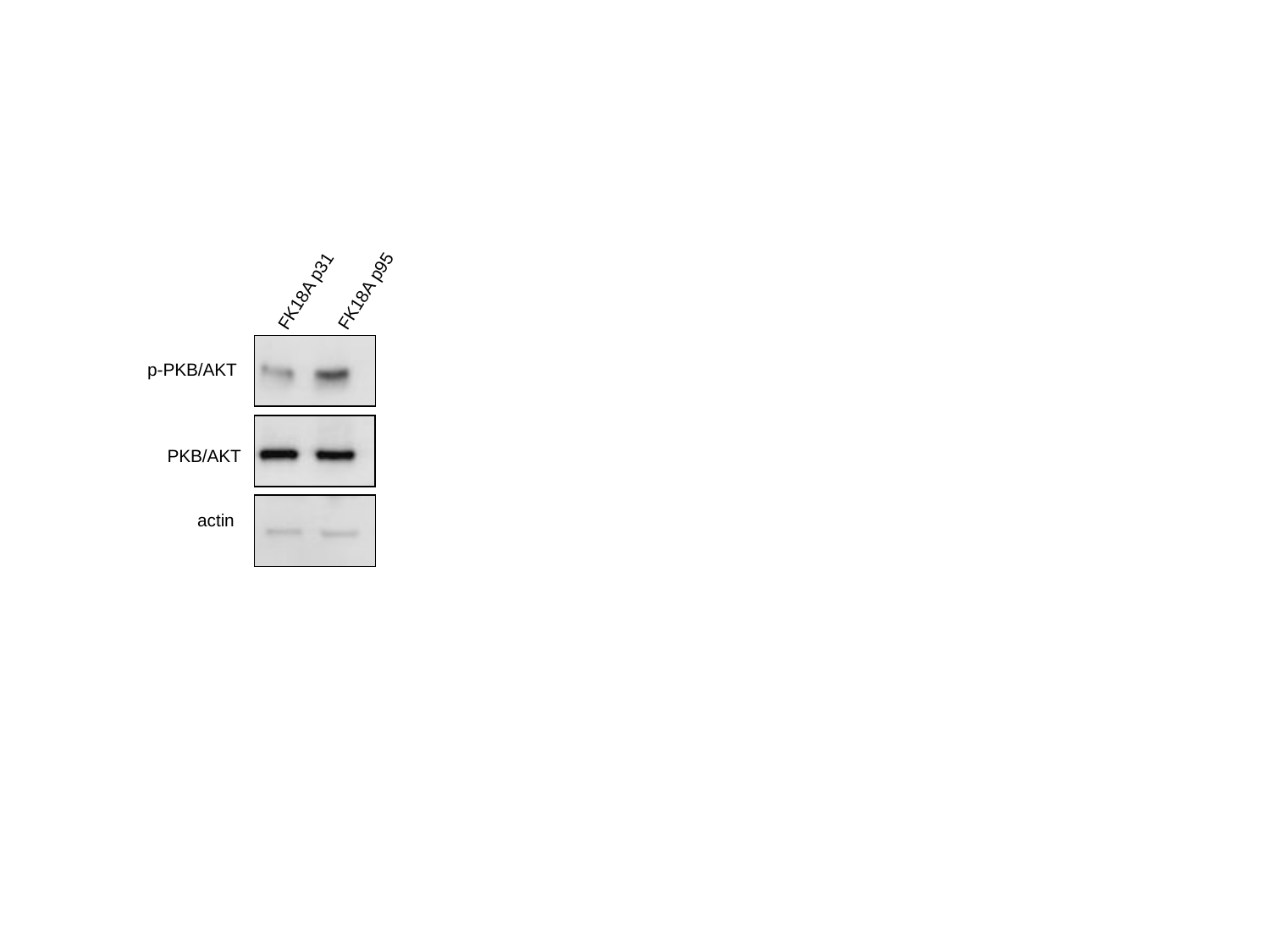

FK18A p31
FK18A p95
p-PKB/AKT
PKB/AKT
actin

Supplement: Additional file 1 — Figure S1. Phosporylated-PKB/AKT and total PKB/AKT protein expression in HPV18-containing keratinocytes. Protein lysates of HPV18-containing keratinocytes at passage 31 and passage 95 were analysed for p-PKB/AKT, PKB/AKT and actin protein expression by Western blot analysis. [file 1476-4598-10-71-S1.PPT]

## Slide 1
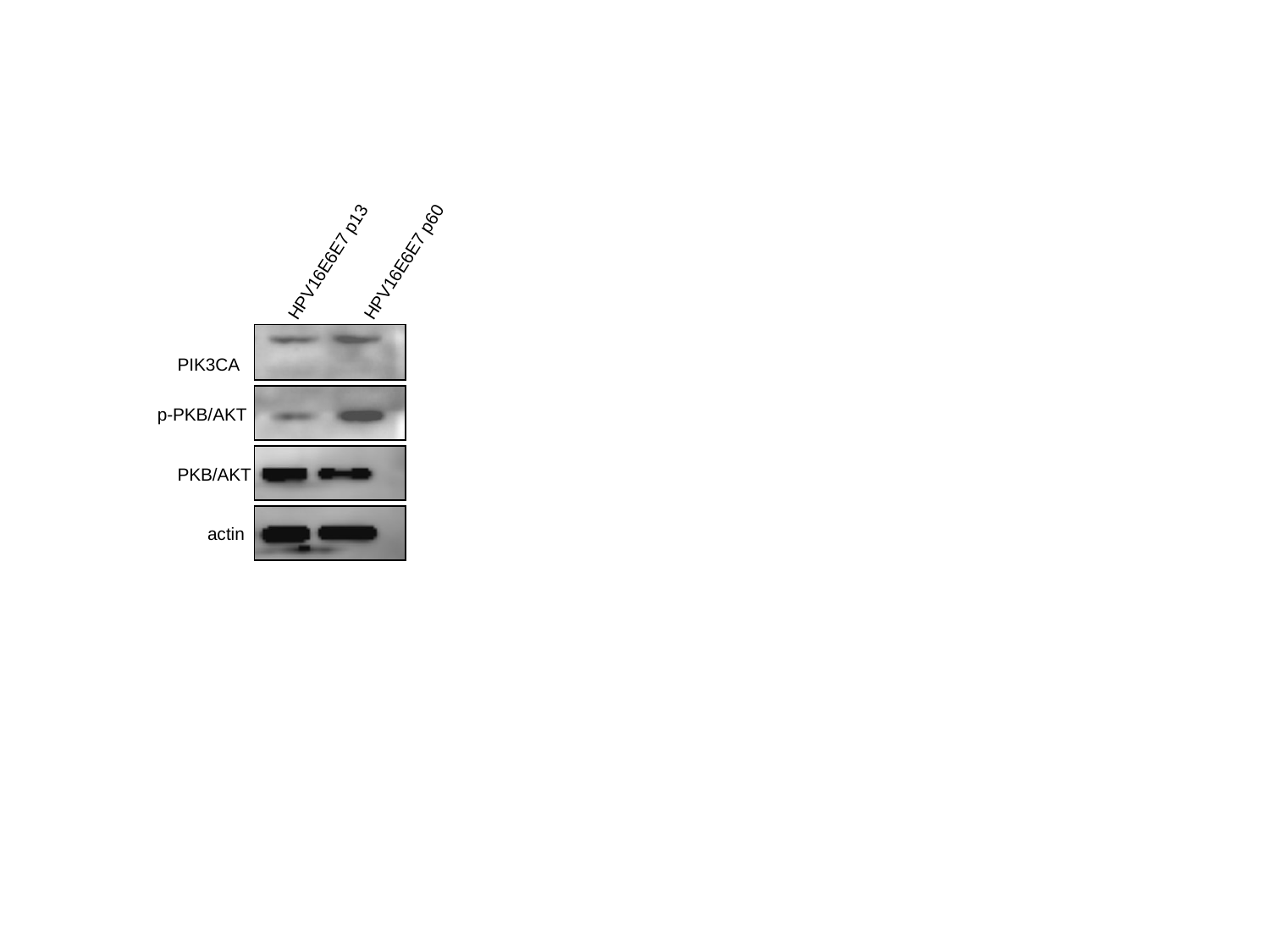

HPV16E6E7 p13
HPV16E6E7 p60
PIK3CA
p-PKB/AKT
PKB/AKT
actin

Supplement: Additional file 2 — Figure S2. PIK3CA, phosporylated-PKB/AKT and total PKB/AKT protein expression in HPV16E6E7 containing keratinocytes from a different donor. Lysates were analysed for protein expression at passage 13 and passage 60 for PIK3CA, p-PKB/AKT, PKB/AKT and actin. [file 1476-4598-10-71-S2.PPT]

## Slide 1
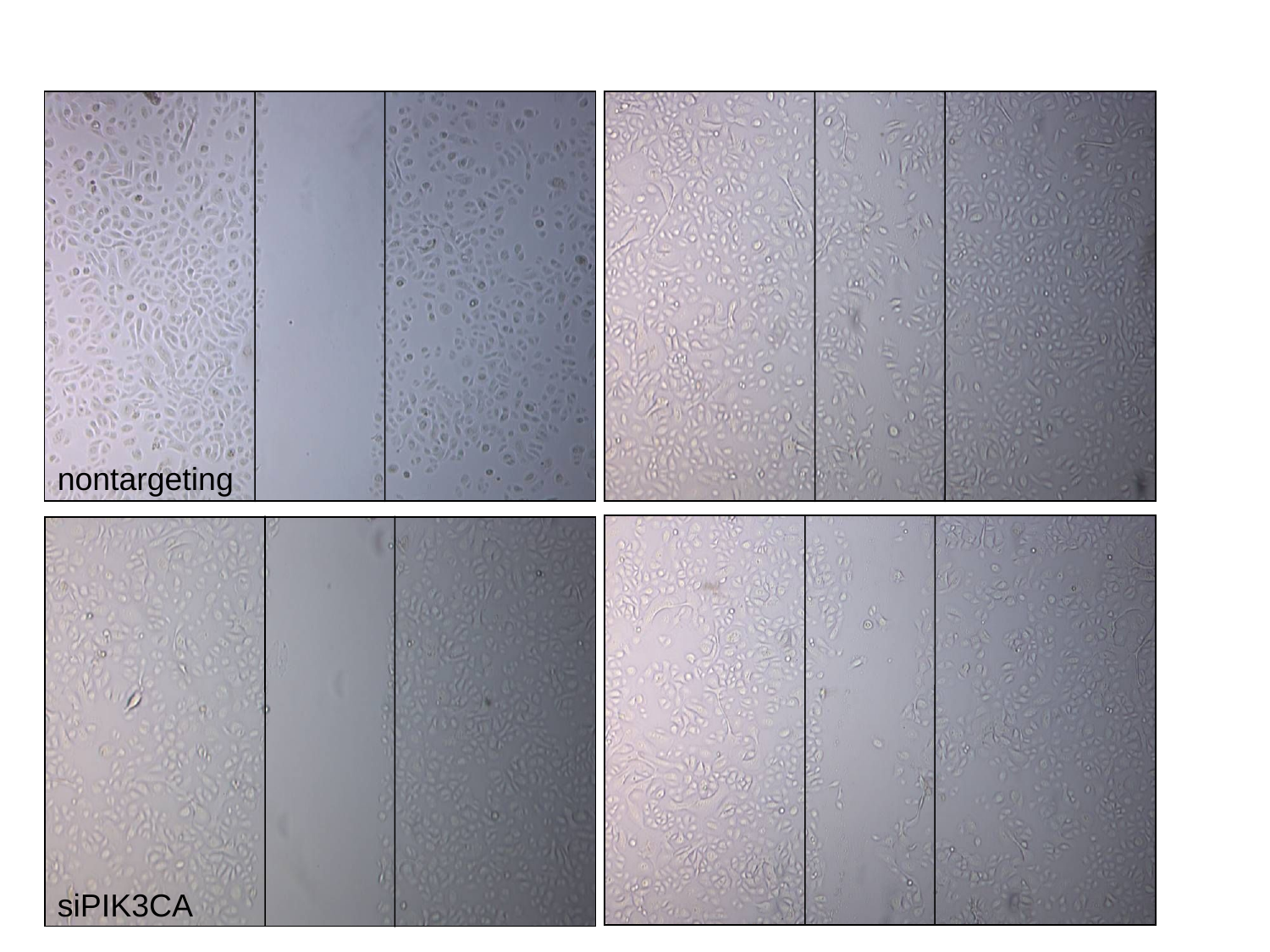

nontargeting
siPIK3CA

Supplement: Additional file 3 — Figure S3. Migration assay of primary human foreskin keratinocytes without and with PIK3CA silencing. Representative pictures of migration assay performed with primary keratinocytes (p5). Upper panel: cells transfected with non-targeting siRNA pool, lower panel: cells transfected with siRNA pool against PIK3CA. Pictures were taken immediately after scratch induction and 24 hours post-induction. [file 1476-4598-10-71-S3.PPT]

## Slide 1
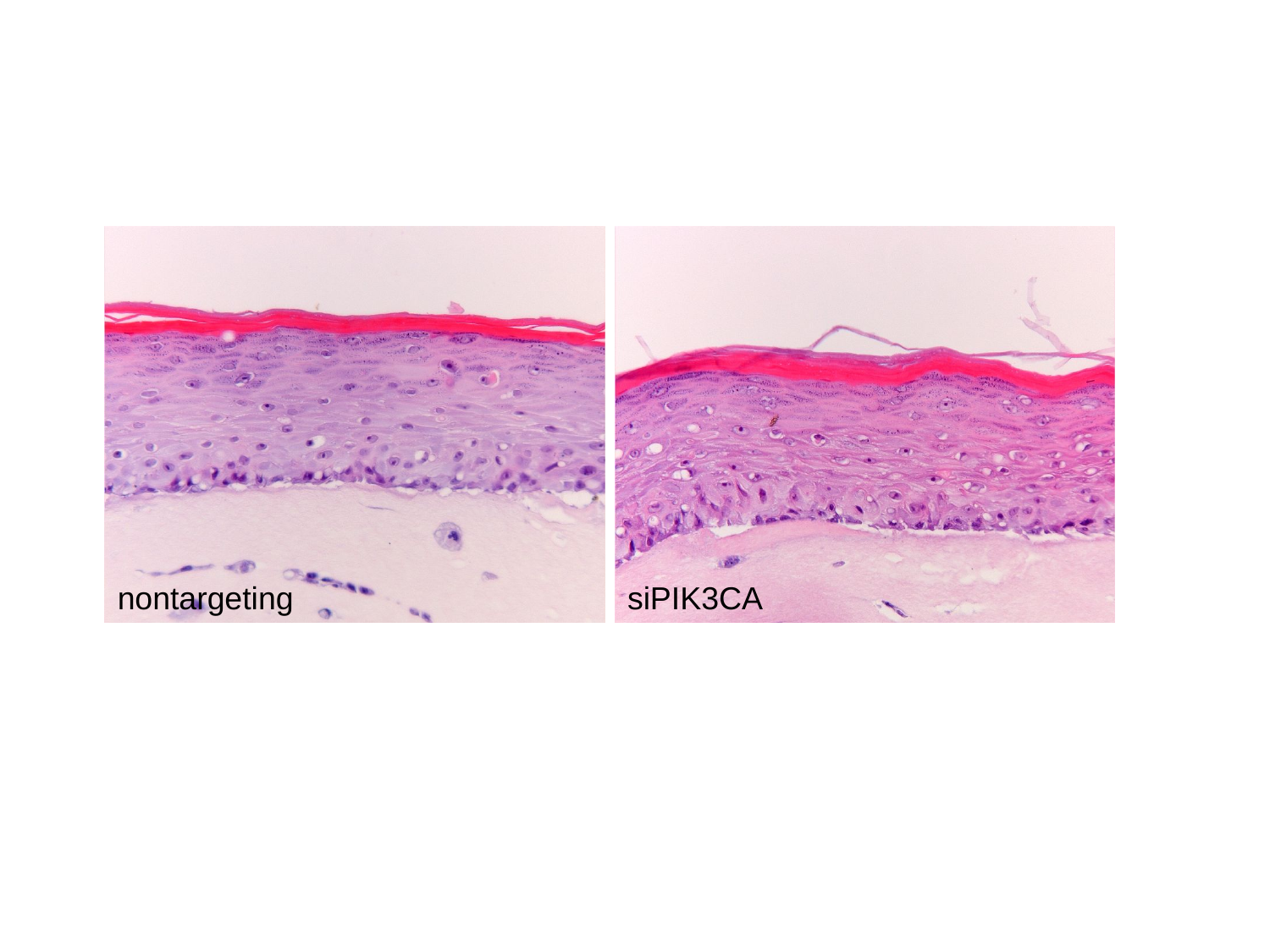

nontargeting
siPIK3CA

Supplement: Additional file 4 — Figure S4. Organotypic raft cultures of primary human foreskin keratinocytes without and with PIK3CA silencing. H&E staining showing raft culture morphology following transfection of primary human foreskin keratinocytes (p3) with non-targeting siRNAs (left) and siRNAs targeting PIK3CA (right). [file 1476-4598-10-71-S4.PPT]
